# COVID-19 street reallocation in mid-sized Canadian cities: socio-spatial equity patterns

**DOI:** 10.17269/s41997-020-00467-3

**Published:** 2021-03-01

**Authors:** Jaimy Fischer, Meghan Winters

**Affiliations:** Faculty of Health Sciences, Simon Fraser University, 8888 University Drive, Burnaby, BC V5A 1S6 Canada

**Keywords:** Active transportation, Built environment, City planning, COVID-19, Policy, Public health, Transport actif, cadre bâti, urbanisme, COVID-19, politique (principe), santé publique

## Abstract

**Intervention:**

Street reallocation interventions in three Canadian mid-sized cities: Victoria (British Columbia), Kelowna (British Columbia), and Halifax (Nova Scotia) related to the COVID-19 pandemic.

**Research question:**

What street reallocation interventions were implemented, and what were the socio-spatial equity patterns?

**Methods:**

We collected data on street reallocations (interventions that expand street space for active transportation or physical distancing) from April 1 to August 15, 2020 from websites and media. For each city, we summarized length of street reallocations (km) and described implementation strategies and communications. We assessed socio-spatial patterning of interventions by comparing differences in where interventions were implemented by area-level mobility, accessibility, and socio-demographic characteristics.

**Results:**

Two themes motivated street reallocations: supporting mobility, recreation, and physical distancing in populous areas, and bolstering COVID-19 recovery for businesses. The scale of responses ranged across cities, from Halifax adding an additional 20% distance to their bicycle network to Kelowna closing only one main street section. Interventions were located in downtown cores, areas with high population density, higher use of active transportation, and close proximity to essential destinations. With respect to socio-demographics, interventions tended to be implemented in areas with fewer children and areas with fewer visible minority populations. In Victoria, the interventions were in areas with lower income populations and higher proportions of Indigenous people.

**Conclusion:**

In this early response phase, some cities acted swiftly even in the context of massive uncertainties. As cities move toward recovery and resilience, they should leverage early learnings as they act to create more permanent solutions that support safe and equitable mobility.

## Résumé

**Intervention:**

Interventions de réaffectation de rues dans trois villes canadiennes de taille moyenne : Victoria (Colombie-Britannique), Kelowna (Colombie-Britannique) et Halifax (Nouvelle-Écosse) en lien avec la pandémie de COVID-19.

**Question de recherche:**

Quelles interventions de réaffectation de rues ont-elles été exécutées, et quelles ont été les tendances en matière d’équité socio-spatiale?

**Méthode:**

Nous avons collecté sur des sites Web et dans les médias des données sur la réaffectation de rues (les interventions ayant élargi l’espace viaire pour le transport actif ou la distanciation physique) entre le 1^er^ avril et le 15 août 2020. Pour chaque ville, nous avons résumé la longueur des réaffectations de rues (en kilomètres) et décrit les stratégies de mise en œuvre et les communications. Nous avons évalué la structuration socio-spatiale des interventions en comparant les différences entre les endroits où les interventions ont été appliquées selon la mobilité, l’accessibilité et les caractéristiques sociodémographiques de la région.

**Résultats:**

Deux éléments ont motivé la réaffectation de rues : la volonté de favoriser la mobilité, les loisirs et la distanciation physique dans les zones densément peuplées et la volonté de stimuler la reprise des affaires touchées par la COVID-19. L’envergure des interventions a varié d’une ville à l’autre; Halifax a allongé son réseau cyclable de 20 %, mais Kelowna n’a fermé qu’un seul tronçon de sa rue principale. Les interventions ont été faites dans les centres-villes et les zones à forte densité de population, à fort usage de transport actif et à proximité des destinations essentielles. En ce qui a trait aux caractéristiques sociodémographiques, les interventions ont eu tendance à être appliquées dans les zones avec moins d’enfants et moins de minorités visibles. À Victoria, les interventions ont été appliquées dans des zones de populations à faible revenu et à proportions élevées de personnes autochtones.

**Conclusion:**

À ce stade d’intervention précoce, certaines villes ont agi rapidement, même en présence d’incertitudes énormes. En s’engageant dans la voie de la reprise et de la résilience, les villes devraient tenir compte de leurs premières conclusions lorsqu’elles commencent à créer des solutions permanentes favorisant une mobilité sûre et équitable.

## Introduction

Physical distancing during the COVID-19 pandemic has disrupted mobility patterns in Canadian cities. With stay at home orders, public transit reductions, and closures of recreational facilities, many people have shifted to active travel for mobility and physical activity. Indeed, the World Health Organization guidelines for getting around during the COVID-19 pandemic stated “whenever feasible, consider riding a bicycle or walking” to help with physical distancing and physical activity (World Health Organization 2020). Increased demand for active transportation has created challenges for physical distancing due to limited public space, particularly on sidewalks, bike lanes, recreational routes, and in dense urban neighbourhoods around everyday destinations.

Governments and public health experts recommend expanding active transportation networks to support physical distancing and physical activity (BCCDC 2020; Government of British Columbia 2020). These expansions, or “street reallocations”, can be considered “population health interventions”—policy actions that may be outside of the health sector but have the potential to impact health at the population level (Hawe & Potvin 2009). Some cities are responding, but the extent and pace of implementation varies from place to place. Street reallocation interventions may be implemented to respond to diverse issues, be it pressure on existing infrastructure, crowding in dense neighbourhoods, narrow sidewalks that do not allow physical distancing, connecting major destinations, or increasing mobility for different populations (Federation of Canadian Municipalities 2020). In July 2020, the Federation of Canadian Municipalities (FCM) released guidelines for COVID-19 street reallocations, with an eye to providing best practices for cities transitioning from temporary to permanent changes (Federation of Canadian Municipalities 2020). These guidelines outline key considerations for selecting an appropriate intervention: filling a gap in the current active transportation network, equity and concentration of priority populations, universal accessibility, proximity to parks and essential destinations (e.g., grocery stores and pharmacies), and access to transit.

In large cities, active travel gets substantial focus, and many large cities are well positioned to respond to shifting needs presented by COVID-19. Vancouver began installing “slow streets” as early as May 2020. These interventions limit motor vehicle traffic volumes on residential streets and greenways to create space for physical distancing while walking and cycling (City of Vancouver 2020). In June, Montreal began reallocating over 300 km of street space to create temporary active transportation circuits of pedestrian and bike paths (Ville de Montréal, 2020), and at the same time Toronto approved a one-year expansion of ~ 25 km of on-street bike lanes (City of Toronto 2020). At the time of writing, many of these interventions were cited as temporary and flexible, although with potential to become more permanent changes.

Mid-sized cities have been less studied than larger cities, despite the fact that most Canadian cities are mid-sized (defined variably as cities with populations of 50,000 to 500,000 people (Flatt & Sotomayor 2016; Winters et al. 2018), or Statistics Canada’s operational definition of “medium population centres” having 30,000 to 100,000 people and densities > 400 people/km^2^ (Statistics Canada 2019)). These cities tend to have fewer resources for active transportation and less complete infrastructure (Flatt & Sotomayor 2016). However, shorter travel distances make active transportation both logical and feasible (Flatt & Sotomayor 2016), and many mid-sized cities have been making bold investments in active transportation in recent years (Winters et al. 2018). Evidence is needed to understand COVID-19 transportation responses across different geographies and active transportation planning contexts. We documented COVID-19-related street reallocations in three mid-sized Canadian cities, Victoria (British Columbia), Kelowna (British Columbia), and Halifax (Nova Scotia), and considered socio-spatial patterning by comparing locations of interventions within each city across area-level mobility, accessibility, and socio-demographic characteristics. These cities are the focus of “Impacts of Bicycle Infrastructure in Mid-Sized Cities Study” (IBIMS), an ongoing population health intervention research study examining how investment in active transportation infrastructure impacts transportation and safety outcomes (Winters et al. 2018).

## Data and methods

We conducted in-depth case studies of street reallocation interventions in three Canadian mid-sized cities to examine a range of experiences in different settings. We documented interventions in Victoria, Kelowna, and Halifax from April 1 to August 15, 2020 and mapped changes against area-level mobility, accessibility, and socio-demographic characteristics. The study areas were selected based on IBIMS protocol (described elsewhere (Winters et al. 2018)). In brief, the geographic boundaries were determined with input from local partners, based on regional travel patterns. The Victoria study area includes the City of Victoria, Township of Esquimalt, District of Oak Bay, and the District of Saanich, which comprise the Greater Victoria urban core. The Kelowna study area is the city administrative boundary, and the Halifax study area includes the Halifax peninsula, mainland Halifax, and Dartmouth.

We used socio-spatial analysis, defined here as an approach that integrates social and spatial data in a geographic information system to identify inequalities in spatial access to resources (in this case, street reallocation interventions) for different socio-demographic groups (Sanchez & Reames 2019). We frame the methodology within the context of equity because it provides a benchmark for assessing the distributional equity of interventions, considering access to essential destinations, mobility, racial and ethnic identity, income, and age. Our unit of analysis was the dissemination area (DA). DAs have populations ranging from 400 to 700 persons and are the smallest geographic area for which all Statistics Canada data are released (Statistics Canada 2018). DAs were ideal from a pragmatic perspective: they are standardized units across cities (whereas definitions of neighbourhoods vary in scale across municipalities), census data and built environment data we use as socio-spatial measures are directly available for DAs, and DAs were sufficiently numerous in our study areas to allow us to look at patterning across quartiles (e.g., Halifax included only 9 neighbourhoods, but 321 dissemination areas). Finally, guidance related to the modifiable areal unit problem suggests that smaller geographic units are better for detecting spatial variation in socio-economic and built environment factors than coarser spatial scales (Biehl, Ermagun, & Stathopoulos 2018). Study area characteristics are in Table 1.

**Table 1:** Land area, population, journey to work mode share, and active transportation infrastructure characteristics (pre-COVID-19) in Victoria, Kelowna, and Halifax

Variable	Victoria	Kelowna	Halifax
Count of DAs	391	163	321
Land area (km^2^)	140.9	213.7	122.4
Population (2016)			
Total population	235,689	128,669	204,927
Population density (people/km^2^)	1672.5	602.2	1674.7
Journey to work			
Walk mode share (%)^a^	13.5	5.6	14.1
Bicycle mode share (%)^a^	8.7	3.7	1.7
Transit mode share (%)^a^	12.9	4.3	17.0
Active transportation infrastructure			
Total bicycle infrastructure (km)^b^	198	243	82
Bicycle infrastructure per person (metres/population)	0.8	1.9	0.4
Total sidewalks (km)^c^	922	400	967
Sidewalk infrastructure per person (metres/population)	3.9	3.1	4.7

^a^Average mode share across study area dissemination areas (Statistics Canada, 2016)

^b^Bicycle infrastructure was obtained from IBIMS city partners and open data and includes linear distance represented by the road centreline (e.g., road km, not lane km, which counts infrastructure separately on each side of the street). Therefore, linear distances listed here will differ from totals cited in bicycling and pedestrian master plans

^c^Sidewalk data were obtained from pedestrian and bicycling master plans (City of Kelowna, 2016; City of Victoria, 2020b; District of Oak Bay, 2015; Halifax Regional Municipality, 2020a; District of Saanich, 2018; Township of Esquimalt, 2019). Lengths are approximate and include linear distance on both sides of the street

### Active transportation context pre-COVID-19

The Victoria study area has 198 km of bicycle infrastructure, including 8 km of protected bicycle lanes in the urban core, and over 900 km of sidewalks. Since 2016, the City of Victoria has been investing in a connected network of all ages and abilities (AAA) bicycle infrastructure which, when complete, will comprise 32+ km of protected bike lanes, shared local street bikeways, and multiuse paths. Initial planning and design involved city-wide consultation with a variety of stakeholders, including neighbourhood associations and members of the public (City of Victoria 2020a). The city is currently completing phase 1, which prioritizes the downtown core and extends into adjacent neighbourhoods. The next phase of development will connect residential neighbourhoods.

Of the study cities, Kelowna has the most bicycle infrastructure (over 240 km), and has ~ 970 km of sidewalks. The city released their *Kelowna on the Move: Pedestrian and Bicycle Master Plan* in 2016, a long-term plan aimed at facilitating active transportation in the region. In recent years, the city has opened a new rail trail and protected bike lanes downtown (City of Kelowna 2016).

At present, Halifax has 82 km of bicycle infrastructure—the least of the three cities—and ~ 790 km of sidewalks. In 2018, the regional council approved the implementation of a AAA bike network outlined in the *Halifax Integrated Mobility Plan (IMP)*, and in 2019 $25 million in joint funding from the federal, provincial, and regional governments was awarded to fast-track network build-out (Infrastructure Canada 2019). Construction has been ongoing since 2018 and is planned to be completed by 2022 (Halifax Regional Municipality 2020a). As in Victoria, extensive public engagement and feedback was incorporated into the planning stage, where various community stakeholders identified route and design options (Halifax Regional Municipality 2020a).

### Data sources

#### Street reallocations

We defined street reallocations as interventions that expand street space for active transportation or physical distancing. Signage and physical barriers were requisite, and street expansions to support businesses (e.g., outdoor patios) were considered only if a substantial amount of the roadway—either a traffic lane or blockwise parking—were reallocated to make space for people. We located interventions through weekly scans of city websites and communications, city social media sites (Facebook and Twitter), and local news articles for the period of April 1 to August 15, 2020. We tracked descriptions, stated strategies (i.e., to support business, mobility, recreation etc.), implementation date, and end date (if applicable) in a spatial database, and mapped the corresponding locations of the interventions using ArcMap 10.7.1 (ESRI Inc. 2019).

#### Socio-spatial measures

Guided by the key considerations in the *COVID-19 Street Rebalancing Guide* (Federation of Canadian Municipalities 2020), we mapped interventions against existing bicycle infrastructure, and population density, mobility, accessibility, and socio-demographic measures at the DA level. All data were available through open data portals and Statistics Canada. Mobility measures include the Active Living Environments (ALE) transit index—a measure of active living friendliness comprised of *z*-scores for intersection, dwelling, points of interest, and transit stop density—from the Canadian ALE database (Can-ALE) (DMTI Spatial Inc. 2016; Ross et al. 2018), and journey to work walk, bicycle, and transit mode share from the 2016 Census (Statistics Canada 2016). Accessibility metrics are from the Statistics Canada Proximity Measures Database (Statistics Canada 2020c) and include proximity to employment, health care, pharmacies, and grocery stores for each dissemination block (DB). Proximity measures are based on the Statistics Canada Business Register, and measure the closeness of a DB centroid to any DB within a predefined network distance. Measures are reported as a normalized index value, where 0 indicates the lowest proximity and 1 the highest proximity in Canada. For employment and health care, proximity measures the closeness of DBs to any DB within a 10-km and a 3-km network driving distance, respectively, and proximity to grocery stores and pharmacies measure closeness to DBs within a 1-km network walking distance (Statistics Canada 2020c). We aggregated proximity scores to the DA level for each indicator and used median values. Socio-demographic measures were included to highlight priority populations and equity considerations. These included median household income, and the proportion who are under age 15, who are over age 65, who identify as a visible minority, who are Black, and who are Indigenous. The proportion of people who are Black is a subset of the visible minority population. Indigenous identity data are collected separately by Statistics Canada and are distinct from the visible minority data. We include explicitly measures for Black and Indigenous as these are populations of interest for equity considerations, given current and historical context in the study cities and in Canada as a whole. The proportions are low (e.g., 1% of the population are Black in the Victoria CMA, 0.7% in Kelowna, and 3.8% in Halifax), and as such there are many DAs with no people who are Black, especially in Victoria (*n* = 235, 60%) and in Kelowna (*n* = 106, 63%), and less so in Halifax (*n* = 77, 23%). There were fewer DAs without people who are Indigenous (< 20% of DAs in Victoria and Halifax, and 4% in Kelowna).

Within each city, we grouped DAs by quartiles for each measure and compared the distribution of street reallocation interventions across quartiles of each mobility, accessibility, and socio-demographic measure. Throughout, quartile 1 reflects areas potentially in need of supports or attention (e.g., higher population density, poorer active living environments, less active transportation, less proximity to essential destinations; for the socio-demographic measures, we inversed the quartiles such that quartile 1 represents higher proportions of visible minorities and Indigenous people, children or older adults, and lower income). Conversely, quartile 4 has lower population density, better active living environments, greatest use of active transportation, closest proximity to essential destinations, and smaller proportions of priority population groups (people who are visible minorities or Indigenous, children and older adults, and those who have lower incomes). These variables are not all normally distributed; in Table 2, we provide quartile cut points for socio-spatial measures in each city.

**Table 2:** Quartile cut points for mobility, accessibility, and socio-demographic measures, by city

	Victoria				Kelowna				Halifax			
Variable	Q1	Q2	Q3	Q4	Q1	Q2	Q3	Q4	Q1	Q2	Q3	Q4
Mobility^a^												
Population density (people/km^2^)	17,341.4	4664.2	3035.2	2032.5	12,363.4	3218.6	2210.5	771.4	50,919.7	5312.6	3247.2	2033.7
Active Living Environment (ALE) (index)	0.0	1.1	2.5	8.9	− 2.5	− 1.5	− 0.5	3.1	− 0.5	0.8	2.3	9.6
Walk mode (%)	4.7	9.3	18.2	54.2	1.0	4.2	9.2	26.1	3.1	7.0	19.4	67.9
Bike mode (%)	4.9	8.6	12.3	29.6	0.0	3.7	5.9	19.4	0.0	3.5	5.5	18.2
Transit mode (%)	8.2	11.8	16.7	40.6	0.0	3.6	6.0	28.6	10.4	16.7	23.1	48.0
Access to essential destinations^b^												
Access to employment (median proximity)	0.04	0.06	0.07	0.13	0.00	0.02	0.03	0.06	0.03	0.04	0.06	0.13
Access to health care (median proximity)	0.01	0.02	0.04	0.11	0.00	0.01	0.02	0.05	0.00	0.01	0.03	0.16
Access to pharmacies (median proximity)	0.01	0.02	0.05	0.06	0.00	0.02	0.03	0.18	0.01	0.02	0.05	0.24
Access to grocery stores (median proximity)	0.02	0.04	0.09	0.46	0.00	0.03	0.05	0.17	0.00	0.03	0.07	0.40
Socio-demographic^a^												
Visible minority (%)	57.4	23.7	15.3	9.2	30.5	12.3	8.2	4.9	70.6	18.7	11.7	6.8
Black (%)	12.0	2.7	1.8	0.0	11.1	2.4	1.3	0.0	61.2	6.4	3.3	0.9
Indigenous (%)	16.7	5.6	3.1	1.7	21.8	8.0	5.3	3.1	19.7	5.1	3.1	1.8
Median household income ($)	46,464	63,616	78,848	158,464	49,920	60,544	80,384	114,432	39,808	51,840	65,664	152,064
Age under 15 (%)	37.4	15.2	12.6	9.4	31.5	17.2	14.2	9.0	38.3	15.6	12.4	9.1
Age over 65 (%)	60.6	26.4	18.8	12.3	82.8	26.2	16.8	12.0	56.2	19.1	14.6	10.2

^a^Mobility and socio-demographic measures are from the 2016 Census (Statistics Canada, 2016) and from the Can-ALE database (DMTI Spatial Inc., 2016; Ross et al., 2018)

^b^Proximity measures are normalized index value ranging from 0 to 1, where 0 indicates the lowest proximity and 1 the highest proximity in Canada, Statistics Canada Proximity Measures Database (Statistics Canada, 2020c)

### Analysis

We described the overarching strategies for street reallocations and their integration with existing active transportation infrastructure. For each city, we summarized total length (km) of street reallocations and described implementation strategies and communications. We mapped intervention locations, and calculated the length of street reallocation interventions within each DA. Where street reallocation interventions coincided with the boundaries of DAs, that length was attributed to each neighbouring DA. We normalized lengths by DA area and present findings as the proportion of distance of total street reallocation interventions, comparing across quartiles of each of the mobility, accessibility, and socio-demographic measures. These charts assess equality in spatial access: if there were equal spatial distribution, each quartile would have 25% of the reallocation interventions. From a mobility justice perspective, a full consideration of equity requires an assessment of the underlying populations’ needs and usage, and may result in targeted interventions for particular populations (e.g., more space in areas with more children). These visualizations are a starting point to assess the distribution of spatial access.

## Results

According to public documents, two main themes motivated street reallocations in the study cities: supporting mobility, recreation, and physical distancing in populous areas, and bolstering COVID-19 recovery for business. Table 3 summarizes the types, extent, and rationale of street reallocation interventions. Of the three cities, Halifax reallocated the most street space (17.2 km) and Kelowna the least (0.7 km). Victoria reallocated 6.4 km. All cities closed streets in core neighbourhoods to create temporary patio space while supporting physical distancing, and Victoria and Halifax expanded sidewalk and street space to support active transportation. The content on cities’ websites did not explicitly mention equity as a consideration in their street reallocation response strategies.

**Table 3:** COVID-19 street reallocations in Victoria, Kelowna, and Halifax between April 1 and August 15, 2020

Intervention	Response strategy	Description	Total street reallocation (km)		
			Victoria	Kelowna	Halifax
Sidewalk expansion	Create space to move	Widen sidewalk by removing parking. Expanded space delineated with bollards, pylons, paint, and signs	1.9	0	0.6
Shared streets	Create space to move; connect active transportation infrastructure	Local (residential) streets closed to through and non-local traffic, and open to local traffic only. Barriers, signage, and pavement markings indicated shared street space	0	0	16.2
Full street closure	Support access to parks and recreation	Streets closed to all public motor vehicle traffic and reallocated to active transportation	3.8	0	0
Temporary patios	Support business and physical distancing	Street/lane closures to create temporary space for seating and gathering while physically distancing	0.7	0.7	0.4
Total street reallocation (km)			6.4	0.7	17.2

Below, city by city, we highlight the strategies cited, the locations of street reallocation interventions and integration with pre-existing infrastructure, and the socio-spatial equity considerations.

### Victoria

#### Interventions and strategies

**Fig. 1:**
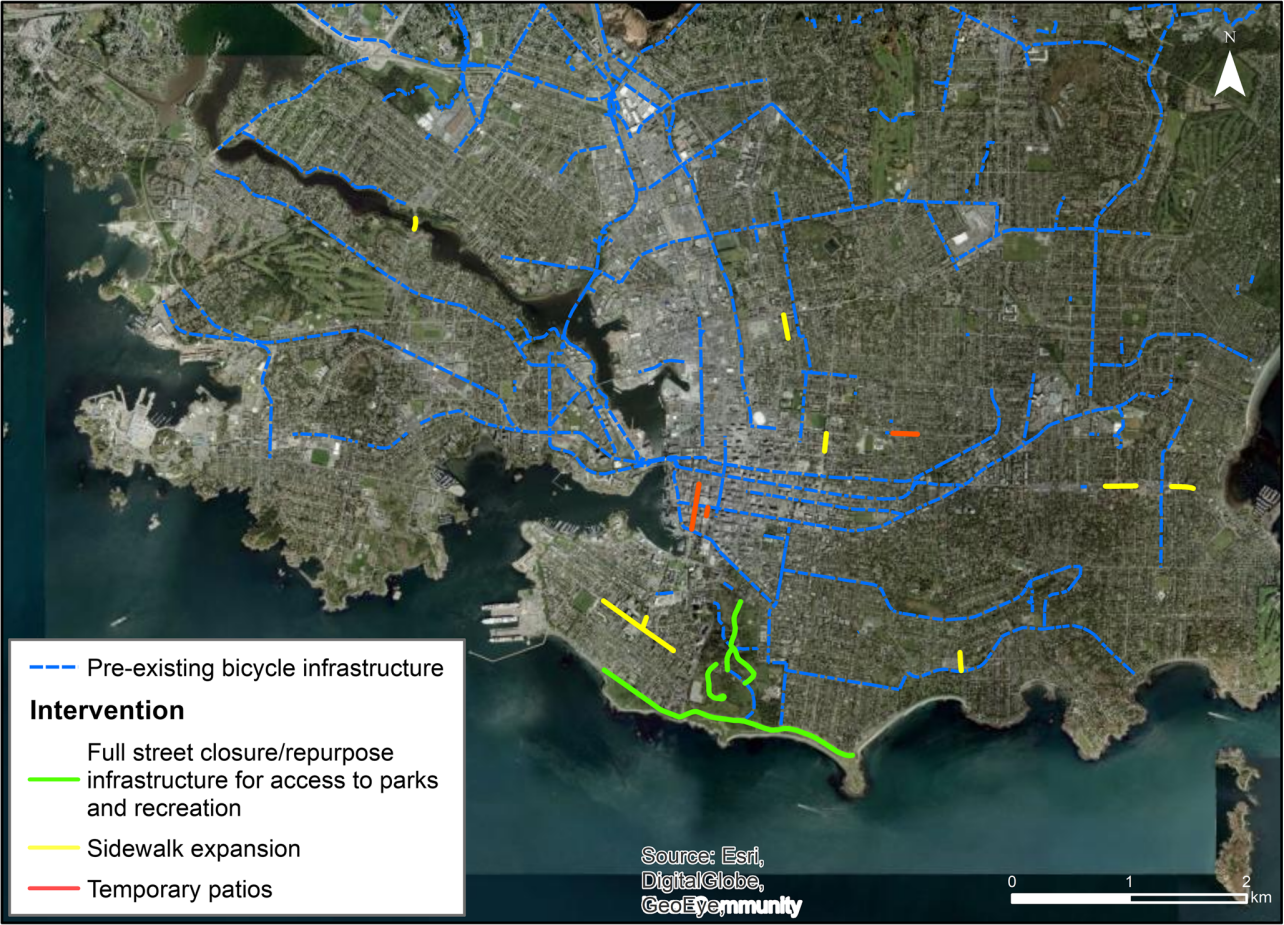
Map of pre-existing bicycle infrastructure and street reallocations in Victoria

In the Victoria area, street space was reallocated for three reasons: expanding sidewalks to create more space for mobility, to support access to parks and recreation, and to support businesses by expanding space for patios (Fig. 1). The response was led by the City of Victoria, who implemented 91% (5.9 km) of street reallocations, by distance. The neighbouring municipality of Oak Bay extended ~ 450 m of sidewalk space along the municipality’s main street, and the regional government expanded ~ 100 m of sidewalk space on the Tillicum bridge to provide additional room for pedestrians during construction. The Township of Esquimalt and the District of Saanich did not make any street reallocations.

The City of Victoria began expanding sidewalk space on April 23, 2020, with the aim to make space for people in dense neighbourhoods to access essential business and services. Space for recreation and access to popular oceanside recreation sites began May 16: in Beacon Hill Park public vehicle access was restricted and streets were designated for active transportation, and along Dallas Road a new protected bike path was repurposed as a multiuse path to provide more space for different modes. In early June, the city closed two main streets to vehicular traffic in the downtown core, reallocating space to businesses opening patios. Street space for business was also reallocated in the Fernwood neighbourhood in early August, and the city is currently accepting applications from businesses for additional reallocations.

In addition to street reallocations, the City of Victoria converted 43 pedestrian activated signals to automated “no-touch” signals, under the strategy to reduce high touch zones. Phase 1 of implementation (May 2020) focused on intersections near grocery stores and hospital and care facilities, and at busy intersections with wide crossings, and phase 2 (late July) prioritized parks, harbourfront and oceanfront routes, and school fields where people may gather. A third phase is in planning stages. The District of Saanich also converted the majority of major pedestrian crossings identified in their active transportation plan to automated signals, but details are unavailable publicly.

Communications for street expansions were disseminated on the *COVID-19 Response and Recovery* and *Build Back* *Victoria *webpages (sites for COVID-19 information for residents and businesses respectively) and the *2020 News *page on the City of Victoria’s website. The city also used their Facebook and Twitter pages to announce street changes, and where parking was removed, delivered letters to residents and businesses along affected routes to notify them. Most changes were reported in local online news. In terms of materials for interventions, sidewalk expansions were delineated with bollards, signs, and paint markings, and street closures were implemented with barricades and signage.

Of the three cities, Victoria had the most existing active transportation infrastructure, and was in the process of implementing a complete, connected AAA bicycle network when the pandemic began. In terms of integration with existing infrastructure, the reallocations supporting recreation connected existing sidewalk and bicycling infrastructure to open space and parks; likewise, the reallocations to support patios and physical distancing in the downtown core were well connected to active transportation infrastructure. Figure 1 shows the interventions and integration with the existing bicycle network.

#### Mobility and accessibility considerations

**Fig. 2:**
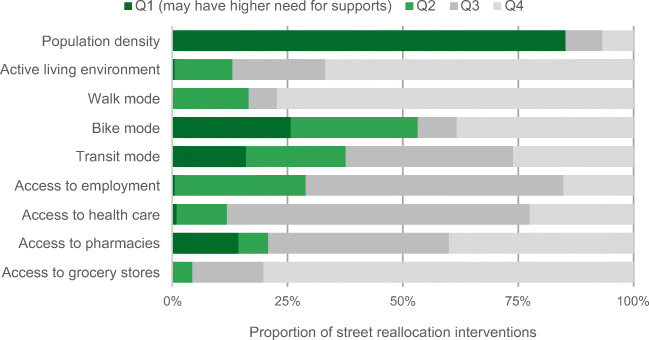
Proportion of total street reallocation interventions in Victoria, across quartiles of mobility and accessibility measures. Quartile 1 reflects areas potentially in need of supports or attention (e.g., higher population density, poorer active living environments, lower mode share, lower proximity to services) and quartile 4 the lowest population density and greatest values for mobility and accessibility measures

We present the relative distribution of street reallocation interventions across quartiles of the population density, mobility, and accessibility measures in Fig. 2. In general in these figures, if there were equal spatial distribution, each quartile would have 25% of the reallocation interventions. Instead, we see the bulk of interventions happen in areas with better active living environments, higher active travel mode shares, and areas with higher population density. Population density presents an opportunity to consider equity, by considering relative need. In more dense areas, people are more likely to have less access to private outdoor spaces, and thus may be in need of access to street space for physical distancing. Notably, all sidewalk expansions were located in higher density neighbourhoods and were sited at “pinch points”, typically in neighbourhood centres with higher levels of essential destinations and pedestrian traffic (Victoria News 2020). In terms of proximity to essential destinations, interventions were in areas with great proximity to grocery stores and health care. In fact, no areas in the lowest quartile of proximity to grocery stores had any street reallocation interventions.

#### Socio-demographic considerations

**Fig. 3:**
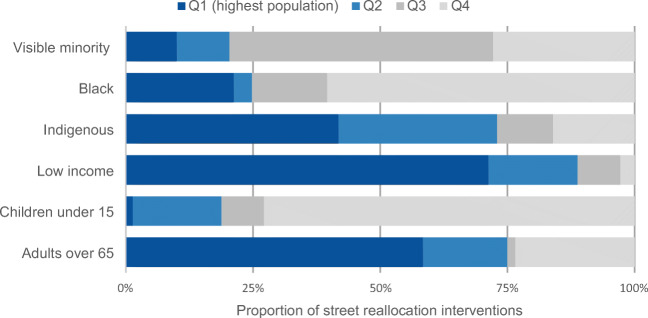
Proportion of total street reallocation interventions in Victoria, across quartiles of socio-demographic measures. Q1 represents DAs with lower income, and higher proportions of visible minority and Indigenous populations, children and youth, or older adults (for the proportion of the population who is Black metric, Q1 comprises 235 DAs that have zero values and other quartiles have ~ 52 DAs)

Figure 3 highlights socio-demographic patterning in the areas with street reallocations. The interventions were predominantly in lower income neighbourhoods, with 71.3% of reallocation interventions in areas falling in the lowest income quartile. In Victoria, these neighbourhoods tend to be closer to the downtown core. However, the interventions tended to be in areas with fewer visible minorities (< 21% of street reallocations (km) were in areas with Q1/Q2 for visible minority measure) and fewer children (< 2% of street reallocations were in areas that had the highest proportion of children (Q1)). In Victoria, children and youth (< 15 years) tend to be concentrated in areas further from the downtown core. Areas with many Indigenous people and older adults were well served.

### Kelowna

#### Interventions and strategies

**Fig. 4:**
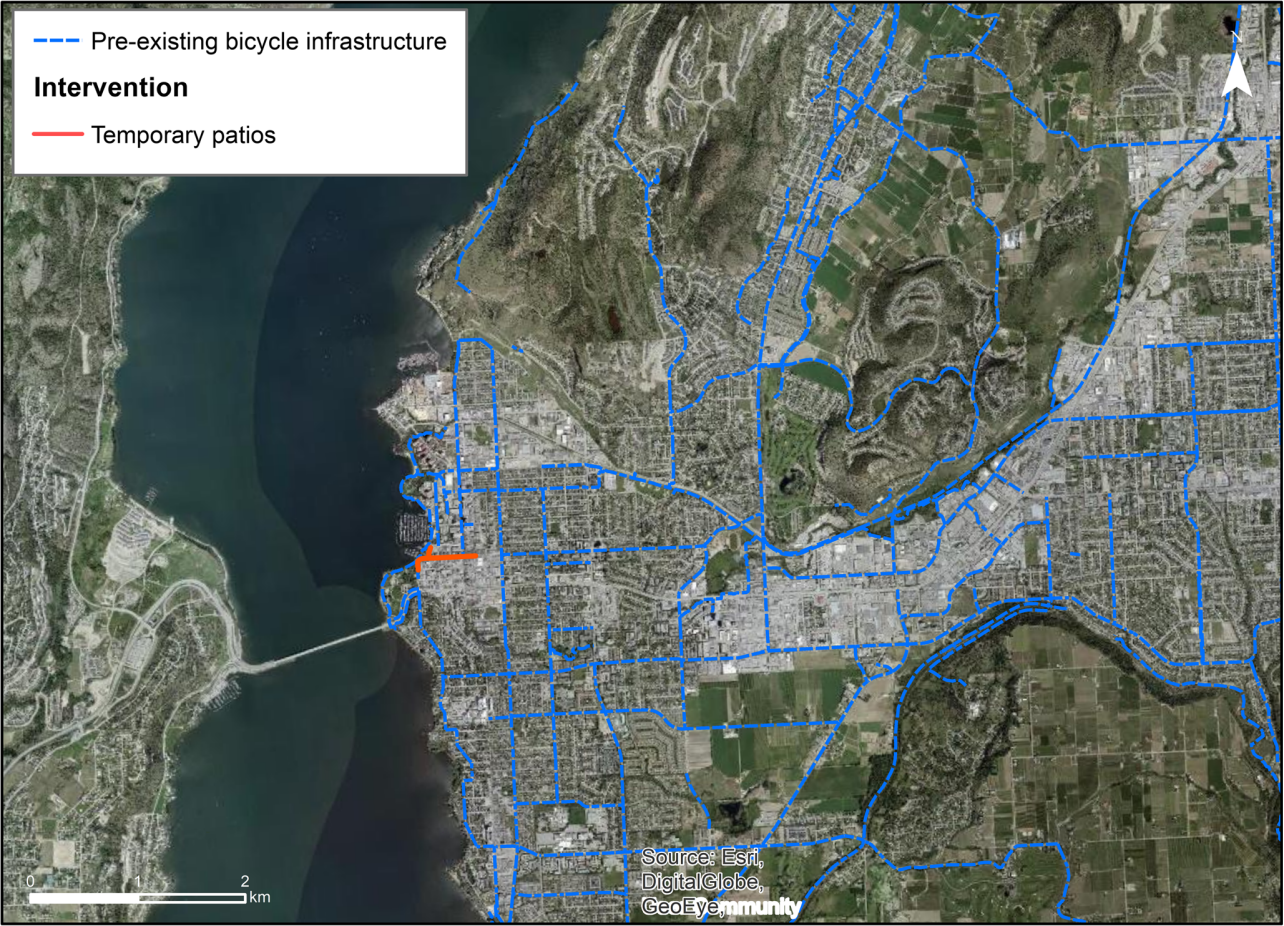
Map of pre-existing bicycle infrastructure and Bernard Ave full street closure for temporary patios and physical distancing in Kelowna

Kelowna allocated just one intervention—a 700 m street closure to motor vehicles along Bernard Ave and short sections of two cross streets—on June 29, 2020 (Fig. 4). The rationale was to support businesses in opening up patios, using road space to allow for physical distancing (City of Kelowna 2020b). Kelowna also automated 36 pedestrian signals in the downtown core. Communications from the city were disseminated on the *News *page of the City of Kelowna’s website, and their Facebook and Twitter pages, and local news covered the closure.

Of the three cities, Kelowna has the most bicycle and the least sidewalk infrastructure and the lowest walking and transit mode shares. The average population density is about half that of the other cities (~ 600 vs 1670 people/km^2^, respectively). The street reallocation intervention was in a strategic location as Bernard Ave is a main street with numerous bars, restaurants, and shops, and is adjacent to the popular lakefront City Park. As shown in  Fig. 4, the street closure connects with sidewalk and bicycle infrastructure.

#### Mobility, accessibility, and socio-demographic considerations

As there was only a single street reallocation intervention in Kelowna, we do not show the relative distribution across socio-spatial measures. Briefly, the intervention was in an area of the city with the highest population density and access to all essential destinations, as well as a supportive active living environment and the highest active transportation mode shares. In terms of social equity, the intervention was in an area with a relatively high proportion of visible minorities and Indigenous people. Like Victoria’s, Kelowna’s downtown core tends to be an area with lower income.

### Halifax

**Fig. 5:**
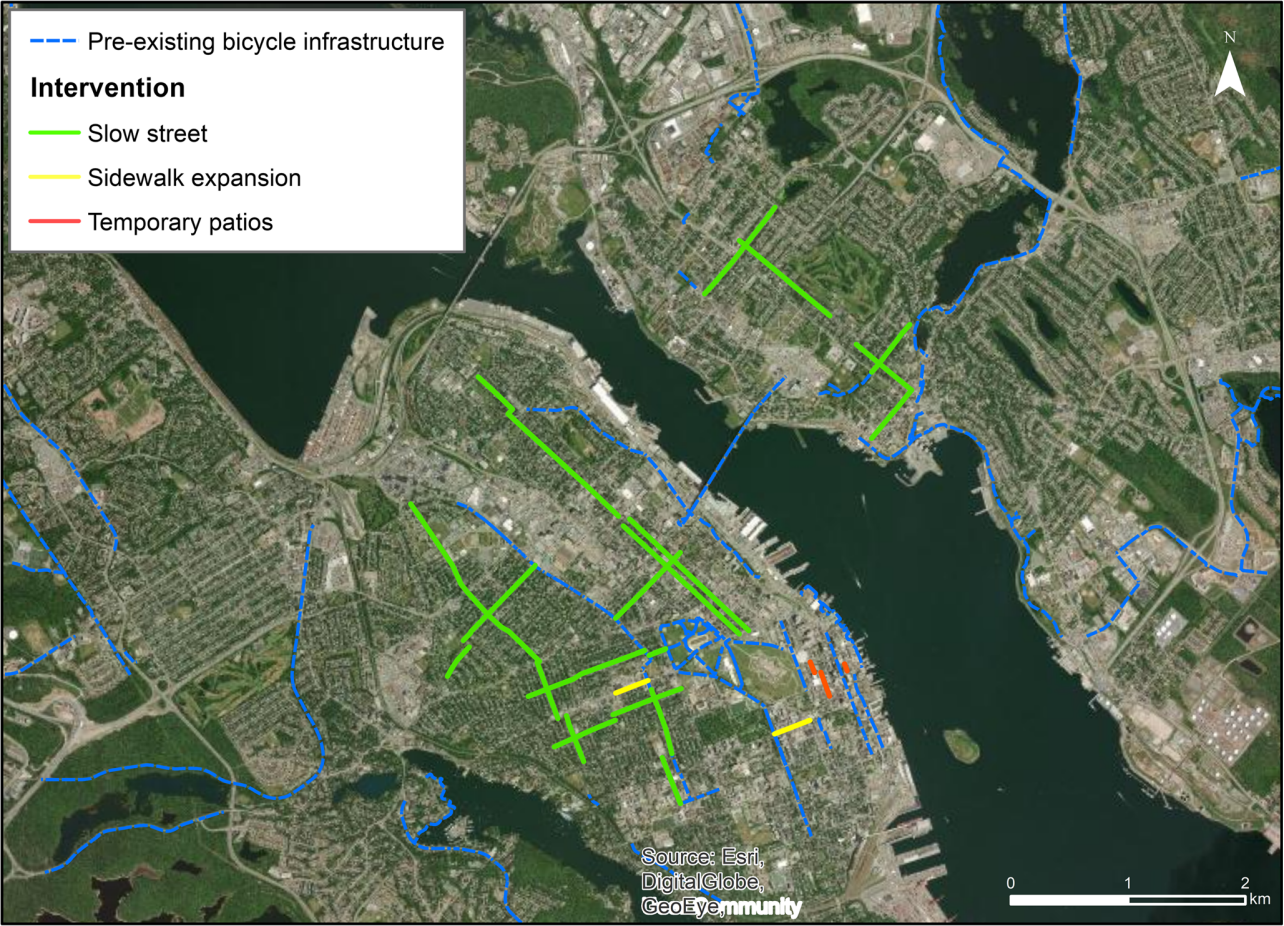
Map of pre-existing bicycle infrastructure and street reallocations in Halifax

Halifax had the most comprehensive response of the three cities, implementing their *Halifax Mobility Response Plan: Streets and Spaces* beginning May 25, 2020. The plan adopted short-, medium-, and long-term actions to adapt public space and transportation networks, which included slow streets, sidewalk expansions, and street closures to support patio expansions and physical distancing (Halifax Regional Municipality 2020b). The region had the least existing active transportation infrastructure of the three cities, and, like Victoria, is actively installing a complete and connected AAA active transportation network. The implementation of slow streets added substantial linkages within downtown Halifax and Dartmouth and connects with some existing infrastructure (Fig. 5).

### Interventions and strategies

The most notable intervention in Halifax was the installation of over 16 km of slow (shared) streets that served to connect neighbourhoods to downtown Halifax and Dartmouth. Additional interventions included ~ 620 m of street space reallocated for sidewalk expansions and ~ 400 m for patio space. Initial siting for slow streets was guided by the *IMP *(~ 50% of slow streets) and additional options were selected and designs adapted based on an older *Active Transportation Priorities Plan* and public crowdsourced feedback through the *Shape Your City* web map (Halifax Regional Municipality 2020b). Slow streets and sidewalk expansions were implemented throughout May and June, and beginning in early July, two streets on the Halifax Peninsula (downtown) were closed or converted to a one-way street to make space for patios.

Sidewalk expansions and slow streets were rolled out under the city’s short-term strategy, with measures intended to be temporary. The plan acknowledges that next steps involve consulting with businesses, advocacy groups, and the public to transition to more permanent (medium-long term) solutions (Halifax Regional Municipality 2020b). In addition to street reallocation, signal timings were altered to reduce wait times at major crossings along 9 major streets.

**Fig. 6:**
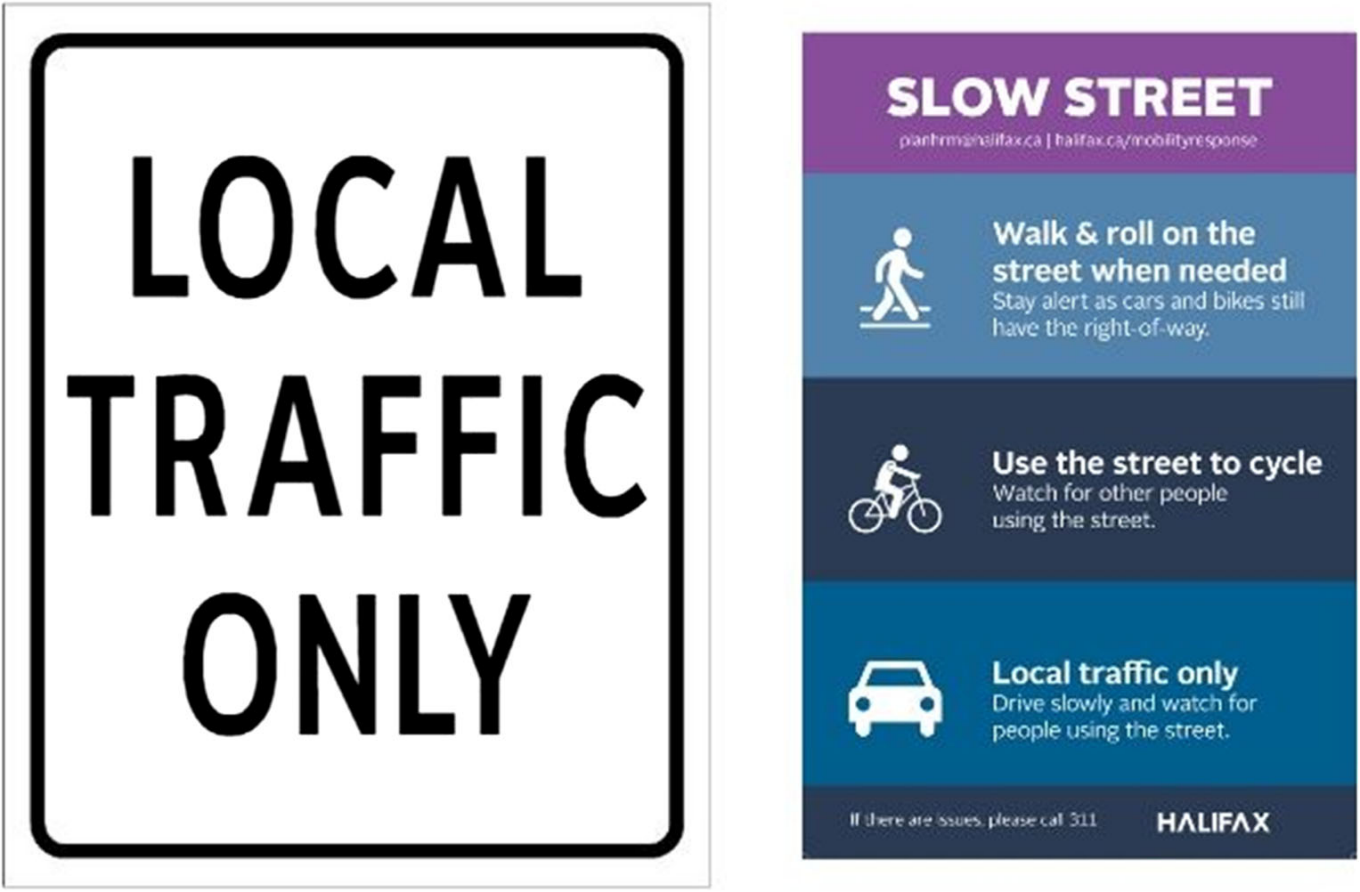
Halifax Mobility Response branding and signage (Halifax Regional Municipality 2020b)

In terms of communication and materials, Halifax adopted branded communications and signage with clear and specific language to raise awareness about interventions (Fig. 6). The city spent $65,000 on extra pylons and barriers to support response (Berman 2020). All interventions were communicated in advance on the *Halifax Mobility Response* webpage and the city integrated public feedback from the *Shape Your City* web map. HRM also released detailed and timely intervention updates on their *News *page, Facebook page, and Twitter. As in the other cities, street reallocations were covered by local media sources.

### Mobility and accessibility considerations

**Fig. 7:**
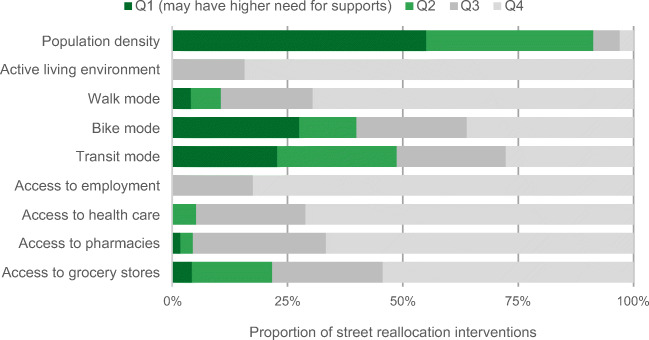
Proportion of total of street reallocation interventions in Halifax, across quartiles of mobility and accessibility measures. Quartile 1 has the highest population density and the lowest values for each mobility or accessibility measure, and Q4 has the lowest population density and highest values for mobility and accessibility

Figure 7 shows the distribution of interventions across mobility and accessibility measures in Halifax. Over three quarters of the interventions were in the areas with the most supportive active living environments (84.2% in Q4); in fact, none were in areas with below median values for active living environment (i.e., Q1/Q2). Interventions were in areas with higher population density and higher use of active transportation, especially walking to work. They were also concentrated in DAs with high accessibility, with upwards of 65% of street reallocations in Q4 for access to employment, health care, and pharmacies, and over 50% for access to grocery stores.

### Socio-demographic considerations

**Fig. 8:**
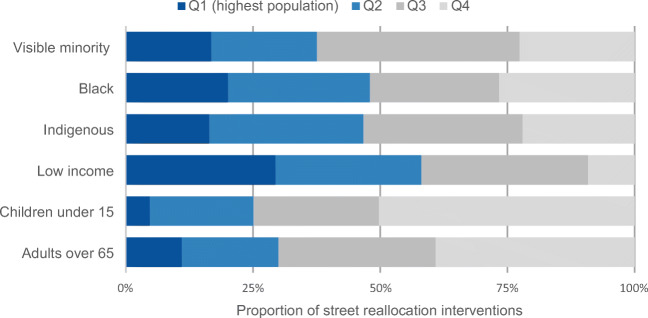
Proportion of total of street reallocation interventions in Halifax, across quartiles of socio-demographic measures. Q1 has the highest proportion of priority populations and Q4 has the lowest

In Halifax (Fig. 8), there was moderate skew toward interventions in areas with lower proportions of people who are visible minorities, Black, or Indigenous (53% to 63% of street reallocation distance in the Q3/Q4 areas for these indicators), but for visible minorities and Black people, this was less pronounced than what was seen in Victoria. The patterning of interventions was fairly balanced across income, although few interventions were in the highest income areas (Q4, highest income, had only 9% of street reallocations). Areas with more children and older adults did not have many interventions (Q1, areas with the greatest proportion of children or the most older adults, had only 4.7% or 11% of interventions, respectively).

## Discussion

Cities have been at the frontline leading rapid responses to adapt to realities of the COVID-19 pandemic. This is an important policy space, as the federal government announces a COVID-19 Resilience Stream under the Investing in Canada Infrastructure Program (Government of Canada 2020a), alongside the Canada Healthy Communities Initiative, which will provide some $31 M to support resilient communities through projects aiming to create safe and vibrant public spaces and improve mobility (Government of Canada 2020b). What are the insights and lessons from these early responses? Here we have analyzed immediate street reallocation interventions in three mid-sized Canadian cities. Mid-sized cities are ready to take action to be sustainable, equitable, and resilient (Evergreen 2018). Yet despite some 8 million Canadians living in cities with populations of 50,000 to 500,000, these cities are routinely overlooked as research and practice focuses on our largest centres.

We have compared actions and strategies of three mid-sized cities during this “early response” phase, which may serve as case studies that resonate with experiences elsewhere. The three cities had different scales of responses, from Halifax adding an additional 20% to their bicycle network, to Kelowna closing only one section along a main street. In terms of mobility and accessibility measures, the interventions tended to be in downtown cores: areas with high population densities, high rates of active transportation, and in proximity to essential destinations. In Halifax, one in five DAs had a street reallocation intervention; in Victoria, one in 14; and in Kelowna, only one in 84. For social equity patterns, the interventions in Victoria and Halifax tended to be in areas with fewer visible minorities. Interventions were often in lower income areas (as these were near downtown cores). People with lower incomes are more likely to be essential workers and may be less likely to be able to work from home; further, they are less likely to own their own cars, and may be most in need of mobility supports (Palm, Allen, & Farber 2020; Statistics Canada 2020b). Finally, interventions were rare in areas with more families. We completed this in-depth analysis in three cities, but expect themes resonate in other places, including larger and smaller centres.

The study cities, like others across Canada, saw shifting active transportation patterns and increased walking and cycling through the spring and summer concurrent with the COVID lockdown (Capital Regional District 2020; City of Kelowna 2020a; Statistics Canada 2020a). Early response interventions included sidewalk expansions, shared streets, full street closures, and temporary patios, but in the available documents it was difficult to assess the relative emphasis on walking, cycling, or business re-opening across early response interventions. We did see cities building off their (pre-COVID) existing active transportation plans, albeit in different ways. The City of Victoria opted not to undertake quick, temporary slow street interventions, but rather to focus energy on advancing their pre-existing plans for their bicycle network, with the AAA treatments as intended. While implementation timeline for the AAA network was initially stalled by COVID-19, council approved designs for the next phase in July and work is slated to begin in the summer (City of Victoria 2020a). In contrast, Halifax moved quickly to put in place temporary slow street interventions along corridors they had already identified as future bikeways in their *IMP*. Both cities already had strong political will toward advancing active transportation goals. Their initial responses differed, but it appeared both were able to capitalize on pre-existing plans to formulate immediate actions that support longer-term mobility goals.

In this early response phase, cities were acting swiftly in the face of massive uncertainties around the pandemic curve, the status of “work-from-home” or “re-start” policies, and public perceptions related to public spaces or public transit. Constraints related to information, time, and resources no doubt played a role in the responses on the ground. Four months into the pandemic the Federation of Canadian Municipalities released the *COVID-19 Street Rebalancing Guide*, a resource compiling best practices in this rapidly unfolding context. The guide highlights three phases for response strategies: “Rapid Response”, “Recovery”, and “Resiliency”. Moving forward through these phases invites some reflection on strategies and impacts thus far. Below we highlight some specific learnings and considerations:

- There is value in capitalizing on existing plans. With the unprecedented need for physical distancing, cities did not have a priori plans or criteria for street reallocations. Not surprisingly, the scale and strategies adopted have varied across cities. However, in our analysis, cities with strong active transportation plans were better positioned for rapid response. Halifax leveraged their recently approved *Integrated Mobility Plan*, implementing some 10 km as slow streets. Capitalizing on this plan carried a benefit of deploying interventions that had already gone through extensive community consultation. In other places, cities have been criticized for failing to seek diverse public input (Thomas 2020).
- Rapid response street reallocations may not support those most in need. Early interventions focused on relieving pressure in the most populated and high activity areas. As we move forward, considering moving toward permanent treatments or implementing new routes, equity-promoting approaches may focus on interventions that connect the areas where priority population groups live to their everyday destinations, including enhanced investments to act on unaddressed need. Careful consideration should also be given to the myriad ways that interventions may impact priority populations. Cities have historically not prioritized engagement with priority populations, a practice that must be changed, particularly as we confront structural racism in urban planning (Pitter 2020). Additionally, as we and others have demonstrated, evaluation that includes spatial analysis is a powerful tool for identifying where priority populations live, and evaluating their access to services and resources (The Centre for Active Transportation 2020). As more cities move to create active transportation plans and access funding to reallocate street space for different mobility options, plans that best serve priority populations and community-specific needs can be accomplished through participatory planning and comprehensive spatial analysis (The Centre for Active Transportation 2020).
- Street reallocations will require more resources. City budgets have been devastated. Some cities may simply have not had funds to undertake reallocations. In other cases, cities actioned quickly, leveraging temporary infrastructure (bollards, signage) which may already have been on hand. Such “lighter, quicker, cheaper” implementations may be sufficient when motor vehicle volumes are reduced/attenuated, but as vehicle volumes bounce back it will be vital that people continue to feel safe and comfortable using active transportation. An outstanding question is to what extent COVID-19 street reallocations will become permanent—and if so, when—given the costs associated with permanent treatments. Hopefully the federal funding programs can help.
- Evaluation is essential. As we move from response to recovery to resilience, much can be learned from the study of early actions as pilot projects. However, few cities, especially smaller centres, seem to have public data on usage patterns, safety, or equity impacts of their street reallocation actions. Such analysis could help develop the case for both locational choices and transitions from temporary to permanent. We hope to see reports of such types of data in the coming months.

## Conclusion

In light of the COVID-19 pandemic, our transportation future is facing much faster and more dramatic change than ever could have been anticipated. The world has seen massive mode shifts and decreased mobility, and a very uncertain future about post-COVID-19 transportation behaviours, as workplaces may support telecommuting, and the public carries concerns around shared transportation. The 2020 Declaration for Resilience in Canadian Cities—signed by political leaders, academics, city planners, and dignitaries—puts out a call to action to repair a history of unsustainable planning, with specific policy actions to responsible land use, decarbonization of the transportation system, and sustainability (Keesmaat 2020). Continued street reallocation interventions, or “rebalancing”, are a tangible step toward this. As cities move toward recovery and resilience, they should leverage early learnings in actions to create more permanent solutions that support safe and equitable mobility.
